# The “Flip-Flap” Technique for Laparoscopic Port-Site Closure—Description of a Novel, Cost-Effective Technique with Review of Literature

**DOI:** 10.1055/s-0041-1731270

**Published:** 2021-07-19

**Authors:** Ajaz Ahmed Wani, Suhail Khuroo, Saurabh Kumar Jain, Vikas Kumar Heer, Deepak Rajput, Shadab Maqsood

**Affiliations:** 1 Department of Surgical Gastroenterology, Shri Mata Vaishno Devi Narayana Superspecialty Hospital, Kakryal, Katra, Jammu and Kashmir, India; 2 Department of Clinical Associate Surgical Gastroenterology, Action Cancer Hospital, New Delhi, India; 3 Department of Surgical Oncology, Shri Mata Vaishno Devi Narayana Superspecialty Hospital, Kakryal, Katra, Jammu and Kashmir, India; 4 Department of General Surgery, AIIMS Rishikesh, Rishikesh, Uttarakhand, India; 5 Department of Radiodiagnosis, Sher-I-Kashmir Institute of Medical Sciences, Srinagar, Jammu and Kashmir, India

**Keywords:** port-site hernia, trocar-site hernia, special type of hernia

## Abstract

**Overview** Laparoscopic approach has changed the face of surgical care offered to patients. Almost all surgical procedures across specialties are now undertaken by the laparoscopic approach. Closure of port sites to prevent trocar-site hernias (TSHs) forms an integral part of the laparoscopic procedure. TSH is an area of preventable surgical morbidity. We hereby report our technique that is easily applicable, simple, safe, and highly cost-effective. It requires no additional instruments or retractors, is easy to learn, and has a very favorable safety profile.

**Materials and Methods** This prospective case series enrolled a total of 454 port-site closures in 255 patients undergoing different laparoscopic procedures over a period of 2 years. The intraperitoneal tissue forceps were used in the reverse direction to lift the fascia up and a right-angled retractor was used to retract back the skin and subcutaneous tissue. The port-site closure is done under vision and no adverse events were reported.

**Results** This technique was used in 454 port sites in 255 patients. No intraoperative incidents were noted. There is no requirement of any specialized instruments or retractors. No additional tissue trauma or dissection is required. There is no extension of operative time. The technique is simple to learn and easy to teach. No bowel injuries or TSHs were reported during a follow-up of 26 months.

**Conclusion** The described technique is easy, simple, cost-effective, and has a good safety profile.

Laparoscopic/minimal access surgery with its notable and proven benefits has changed the face of surgical care offered to patients. Almost all surgical specialties are routinely employing the laparoscopic route to perform procedures that were previously done by the open approach. This emergence of laparoscopic approach has brought to the forefront an entirely different set of complications. Many of these complications are areas of potentially avoidable surgical morbidity.

Trocar-site hernias (TSHs) are a special type of hernias which are specific to the laparoscopic route. Crist and Gadacz, defined TSH as hernias developing at the sites of cannula insertion sites. 1 The terms TSH, port-site hernia, and port-site incisional hernia are often used interchangeably. The presence of a peritoneal covering/hernia sac is not a prerequisite to define a TSH. 2

The incidence of TSH cited is highly variable among various series in the literature. The incidence of TSH varies between 0.15 and 1.8% 3 4 following laparoscopic cholecystectomy. The Dutch data from 2004 revealed a symptomatic incisional hernia rate of 0.61% following laparoscopic appendectomies and a TSH rate of 0.8% following laparoscopic cholecystectomy. 5 Boldó et al reported a TSH rate of 22% following laparoscopic ventral hernia repair. 6 Paya et al reported a TSH rate of 1% from their retrospective review of 293 laparoscopic procedures in a 4-year period from a pediatric surgical unit. 7

The development of TSH depends on both patient-related factors and the surgical technique employed for port-site closure. Many methods of port-site closure are currently employed. However, the ideal method of laparoscopic port-site closure continues to be debated upon ( Table 1 ).

**Table 1:** Distribution of cases

Procedure	Number	Number of ports
Lap cholecystectomy	156	312
Lap appendectomy	43	86
Lap CAPD insertion	24	24
Lap ventral hernias	23	23
Diagnostic laparoscopy	6	6
Lap-assisted colon resections	3	3

Abbreviation: CAPD, continous ambulatory peritoneal dialysis; Lap, laparoscopic.

The aim of this study was to describe our experience with this novel and simple port-site closure technique in light of the available literature.

## Technique Description

**Fig. 1:**
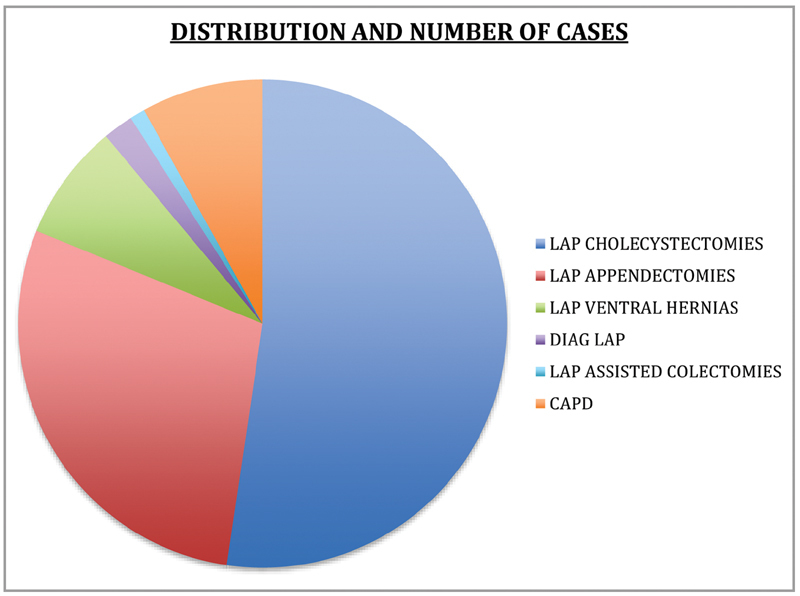
Distribution of cases.

**Fig. 2:**
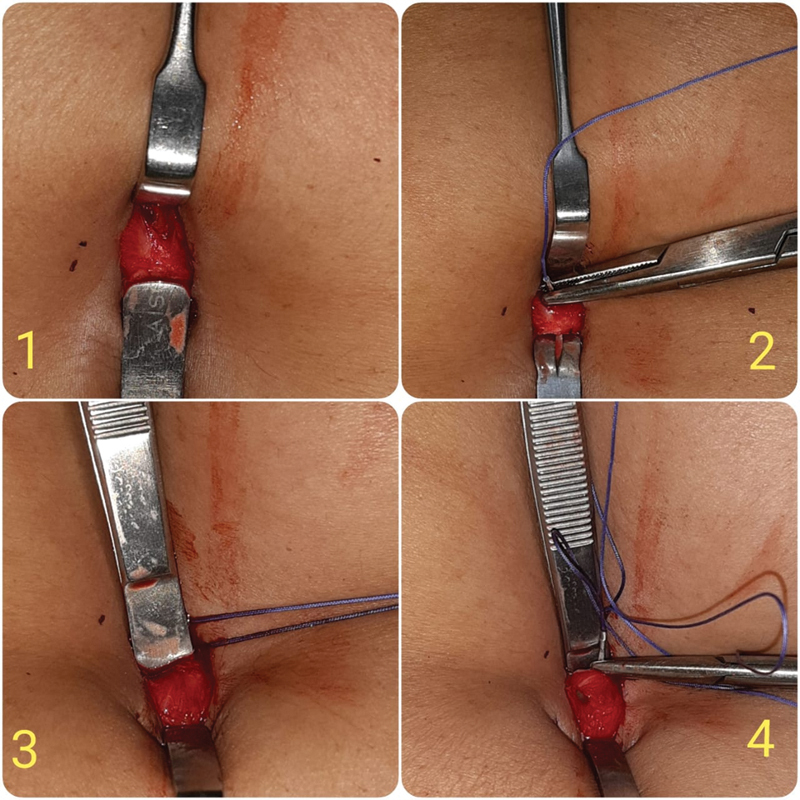
Flip flap.

The surgical procedure requires a surgeon and a single assistant ( Fig. 1 ). Standard operative room equipment is required. There is no need for any specialized instrument or retractor. After deflating the pneumoperitoneum, the surgeon inserts a tissue holding forceps in the reverse direction into the peritoneal cavity ( Fig. 2 , Picture 1). The skin and subcutaneous tissue are retracted back by a right-angled retractor to expose the fascia ( Fig. 2 , Picture 2). Using the peritoneal tissue forceps as a guard, a good tissue bite is taken ( Fig. 2 , Picture 2). The intraperitoneal surface forceps is then “flipped” by 180 degrees to repeat the steps on the other side of the incision ( Fig. 2 , Pictures 3 and 4). Depending on the defect dimension, one or two interrupted sutures are taken. If the fascia is not properly visible, the skin incision can be extended by few millimeters on either side to provide enough glide to expose the sheath.

## Results

The described technique was employed for closure of 454 (10 mm) port sites in 255 patients. The median body weight was 83 kg and the median body mass index was 27.7 kg/m ^2^ . There were no infections in the port sites that were closed by this technique. No TSHs have been observed for a mean follow-up of 26 months. There have been no adverse intraoperative events by the use of this technique. Both operating surgeons are routinely using this technique for port closure in their cases since the past 2 years.

## Discussion

TSH is a known complication of laparoscopic surgery. It is an area of preventable surgical morbidity. The earliest description of a TSH has been attributed to Fear 8 from his review of laparoscopy in gynecological procedures. Maio and Ruchman 9 reported the first case of small bowel obstruction complicating a TSH. Schiff and Naftolin reported two cases of small bowel herniation following laparoscopic procedures. 10

The etiology of TSH is multifactorial and depends on both patient- and surgical technique-related factors. Trocar size, trocar design, trocar location, technique employed for creating pneumoperitoneum, and postoperative port-site infection are some important technical factors responsible for development of TSH.

Trocar designs have undergone a technological evolution. The earliest trocars had pyramidal tip configuration with cutting edges. Disposable-shielded trocars with spring load mechanisms were the next-generation trocars to be introduced. The newer conical tip, dilating (radial or axial) bladeless trocars create a smaller abdominal port diameter, and they pierce tissue by splitting rather than by cutting the muscles. 11 Optical trocars consisting of a crystal tip along with a zero-degree telescope are among the most recent trocars available. Up-gradation of trocar design to minimize their deleterious effect is an emerging field in the surgeon–industry relationship.

As per available literature, majority of cases of TSH occurred with port size of 10 mm ports. 2 However, TSH has also been reported with a trocar size as small as 3 mm. 12 The American Gynaecologic Laparoscopic Surgeons survey cited that 86.3% of TSH occurred with trocar diameter of 10 mm. 13 Lambertz et al, in one of the largest series of TSH of 54 cases, cited that TSH occurred in 96% after the use of trocars with 10 mm and larger diameter. 14 Consequently, it is recommended to close all port sites > 10 mm routinely. 2 13 14 The closure of port sites with smaller diameters should be considered in pediatric patients, after excessive port manipulation and when predisposing risk factors are present. 15 The ideal method of port-site closure, however, remains debatable.

Periumbilical midline trocar sites carry the highest risk for development of TSH, secondary to inherent tissue weakness at this level. Ports in the lateral abdominal wall are covered by anterior and posterior sheaths with muscle layers in between and are consequently less susceptible for development of TSH. Upon trocar removal, the anterior and posterior sheaths approximate in a “shutter mechanism” decreasing the potential of a TSH. 16 The same principle is the basis of creating oblique “Z tracks” which pierce the anterior and posterior sheaths at different levels providing good overlap upon trocar retrieval.

Malnutrition and chronic catabolic states such as liver failure/cirrhosis, renal failure, and diabetes predispose to a higher risk for development of TSH. 1 17 18 Conditions that lead to raised intra-abdominal pressure such as obesity and chronic obstructive airway disease (COAD) are also related with higher incidence of TSH.

The time of presentation of TSH can vary. It has been reported to occur even in the first few days after surgery. 19 Patients harboring a TSH can be asymptomatic or can present with features of small bowel obstruction and/or even strangulation. Since the diameter of the port site is small, only a part of the circumference of the bowel wall gets trapped. Hence a Richter's type hernia formation takes place, which may present insidiously and in an atypical manner warranting a high index of suspicion especially initially when a prominent bulge or swelling is absent. 17 Besides in the early postoperative period, any port-site swelling can also be erroneously labeled as a seroma or hematoma and features of small bowel obstruction can be falsely attributed to paralytic ileus. In doubtful cases, cross-sectional imaging such as contrast-enhanced computed tomography or magnetic resonance imaging can help refine the diagnosis and guide further treatment. 18

Based on the defect configuration and the timing of presentation, Tonouchi et al 2 have proposed a classification system for TSHs. The three types comprise:

1. Early type
2. Late type
3. Special type.

Adequate approximation of tissues is the cornerstone to prevent any fascial disruption, which may act as a prelude for development of TSH. Inadequate approximation, postoperative infection, or suture disruption may lead to development of a TSH. 8 Apart from the commonly used hand suture technique, 29 other methods have been described for laparoscopic port closure. 20 21 Instruments as diverse as the commonly used spinal needle, hypodermic needle, aneurysm needle, vein catheter, angiocath needle set, Deschamp's needle, and Berci's needle have been described for closure of the fascial defect. On the other extreme, many new instruments such as Maciol Suture needle set, Endo Close suture device, Carter-Thomason device, Gore-Tex suture passer, and Tahoe surgical instrument ligature device have also been described for their use in closure of laparoscopic port-site closure.

Shaher 20 classified port closure techniques into following three categories:

1. Techniques that use assistance from inside the abdomen and require two additional ports
2. Techniques that employ additional extracorporeal assistance and require one additional port
3. Closure techniques that can be performed with or without visualization and require no additional ports.

First and second groups of techniques require the presence of pneumoperitoneum and the closure is always performed under direct visualization.

The management of TSH depends on the type of hernia and the mode of presentation. Primary repair and mesh reinforcement of the hernia defect (open/laparoscopic) are the two most commonly used modalities. In early type and special type of TSHs, 2 prompt surgical management is warranted to prevent strangulation. Late-onset type TSH 2 needs reduction of the contents followed by fascial closure either by open or laparoscopic approach. Laparoscopic approach also allows for visualization of the herniated contents and the possibility of intraperitoneal on-lay mesh repair. Laparoscopic approach may require creation of additional ports, which are potential sites of further hernia formation.

The available literature regarding the management of TSH is scarce. Treatment is guided by studies with limited sample sizes and anecdotal available case reports. The fascial defect dimension that acts as the “ borderline “ between suture and mesh repair has not been properly defined. In one of the largest series of TSH till date, Lambertz et al 14 proposed a fascial defect size of 2 to 3 cm to be the crossover margin between primary repair and mesh reinforcement. The need of the hour is prospective studies on a larger scale enrolling higher number of patients to formulate guidelines for prevention and treatment of TSH.

The described technique belongs to category 3 as per Shaher's classification as there is no need for any additional port or pneumoperitoneum. It is simple to learn and easy to teach and hence easily reproducible. The technique is highly cost effective as there is no need for any additional or costly instruments. The instruments required are easily available universally. No additional tissue dissection or trauma is caused. We recommend this technique to be used in all laparoscopic port-site closures in nonobese patients.

## Conclusion

TSH is a known complication of laparoscopic surgery. It is an area of preventable surgical morbidity. With the universalization of laparoscopy, the incidence of TSH is bound to increase. The described technique is simple, safe, easy, and cost-effective. We recommend this as the technique of choice to be employed in all laparoscopic port-site closures in nonobese patients.
